# Understanding the Role of Carbon Fiber Skeletons in Silicone Rubber-Based Ablative Composites

**DOI:** 10.3390/polym14020268

**Published:** 2022-01-10

**Authors:** Yuan Ji, Shida Han, Zhiheng Chen, Hong Wu, Shaoyun Guo, Ning Yan, Hongyan Li, Tao Luan

**Affiliations:** 1 The State Key Laboratory of Polymer Materials Engineering, Polymer Research Institute of Sichuan University, Chengdu 610065, China; JiYuan_scu@163.com (Y.J.); sdhan1996@163.com (S.H.); chenzh104@163.com (Z.C.); nic7702@scu.edu.cn (S.G.); 2 Xi’an Modern Chemistry Research Institute, Xi’an 710065, China; chipsme@126.com (H.L.); yimubath@163.com (T.L.)

**Keywords:** silicone rubber, ablation, carbon fibers (CFs), skeleton

## Abstract

At present, silicone rubber-based ablative composites are usually enhanced by carbon fibers (CFs) to protect the case of solid rocket motors (SRMs). However, the effect of the CFs’ length on the microstructure and ablation properties of the silicone rubber-based ablative composites has been ignored. In this work, different lengths of CFs were introduced into silicone rubber-based ablative composites to explore the effect of fiber length, and ceramic layers of various morphologies were constructed after ablation. It was found that a complete and continuous skeleton in ceramic layers was formed by CFs over 3 mm in length. In addition, the oxyacetylene ablation results showed that the linear ablation rate declined from 0.233 to 0.089 mm/s, and the maximum back-face temperature decreased from 117.7 to 107.9 °C as the length of the CFs increased from 0.5 to 3 mm. This can be attributed to the fact that successive skeletons concatenated and consolidated the ceramic fillers as well as residues to form an integrated, robust, and dense ceramic layer.

## 1. Introduction

Solid rocket motors (SRMs) are mainly used as propulsion for launch vehicles owing to their stability, reliability, and, especially, high transmitter mobility [1,2,3,4]. During the operation of SRMs, high-temperature and high-speed heat flux with particles is produced in the combustion chamber, which may lead to the destruction of the combustion chamber case, resulting from complex ablation including thermochemical corrosion, particle concussion, gas shear, and external pressure [5,6,7,8]. To keep the motor running, the thermal protection materials (TPMs) are positioned between the propellant grain and the case, which can not only resist ablation but also buffer the generated stress and strain [6,9]. Due to the backbone consisting of only –Si–O– bonds, silicone rubber possesses good thermal stability, favorable flexibility, and low thermal conductivity [10,11], and it can form pyrolysis products in an ablative environment that resist high-temperature and oxidizing gases [12]. Therefore, silicone rubber is suitable as a matrix for TPMs.

The ablative properties of silicone rubber-based ablative composites (i.e., resistance to thermochemical corrosion and mechanical scouring as well as thermal stress caused by high-temperature and high-speed heat flux) are mainly determined by the structure of the ceramic layer. When silicone rubber is heated, the temperature increases until reaching the pyrolysis temperature, and then it initiates to release gaseous products, leaving porous ceramic residues (i.e., ceramic layers) [13]. Then, the ceramic layer, as a barrier against ablation, proceeds to absorb heat until it reaches the temperature at which it is oxidized and melted as well as sublimated or mechanically removed by shear force [14,15]. However, the residual yield of silicone rubber is low (9 wt% at 800 °C in argon), and the formed ceramic layers are weak and brittle [13,15]. In recent years, high melting point fillers, such as ceramic fillers [14,16,17], high residual yield resins [18,19,20], flame retardants [10,21,22,23], and fibers [24], have been intensively investigated to improve the content and compactness of the ceramic layer. Usually, fibers are used to withstand mechanical erosion (including the impact of particles, thermal stress, and internal pressure) for increasing the retention time of the ceramic layer [25,26,27]. Among the fibers used as reinforcement, carbon fibers (CFs) with a graphitization structure have the characteristics of excellent thermal stability and strength. Especially in high-temperature environments, its high-strength, non-melting, and non-combustibilty can effectively carry externally transmitted loads [28]. Consequently, CFs are widely used to improve the ablation resistance of silicone rubber-based ablative composites.

Many studies have confirmed the capability of CFs to improve the ablation properties of composites. On the one hand, CFs promote the formation of a char layer with good strength, and a dense and complete surface is formed by CFs [29]. On the other hand, reciprocity between the ablation layer and the composite is strengthened due to the addition of CFs, which makes it difficult to be peeled off during ablation [30,31]. Since CFs make an important contribution to ceramic layer structure and the ablative performance of silicone rubber-based composites, fiber length is bound to have a major impact on the ablative behavior. However, due to the fact of their brittleness, CFs are easily destroyed by shear force in the process of materials preparation (two-roll mix) and cannot maintain their original length. The effect of the actual length of CFs on the microstructure and properties of silicone rubber-based ablative composites has been neglected. Therefore, the correlation between the actual length of CFs and composites performance needs to be clarified for efficient utilization of CFs and designing of the structure of the ceramic layer to improve the ablative properties of silicone rubber-based ablative composites.

In this work, to avoid the breakage of CFs induced by strong shear force and to introduce other factors, a solution mixing approach was adopted to obtain silicone rubber-based ablative composites enhanced by CFs with different lengths, and the effects of fiber length on ablative resistance, ceramic layer morphology, and thermal properties were investigated systematically. The relationship between ablative performance and a ceramic layer structure was evaluated and a related ablation mechanism was proposed.

## 2. Materials and Methods

### 2.1. Materials

The polydimethylsiloxane (PDMS) and polydimethylphenylsiloxane (PMPS) were supplied by the Shanghai Resin Co., China. The average molecular weights were approximately 600,000 and 700,000, respectively. The vinyl contents of the two rubbers were both 0.15%, and the phenyl contents of PMPS were 10.6%. Fumed silica (average particle size: 5 μm) was supplied by Shijiazhuang Ruituo Chemical Technology Co., Ltd. (Shijiazhuang, China). CFs with a length of 6, 3, 1, and 0.5 mm were supplied by Shanghai Lishuo Composite Technology Co., Ltd. (Shanghai, China). 2, 5-dimethyl-2, 5-di-(tert-butylperoxy)hexane (DBPMH) was used as a vulcanizator supplied by Shanghai Sendychem Co., Ltd. (Shanghai, China). ZrB_2_ (average particle size: 5 μm) and B_4_C (average particle size: 3–5 μm) were supplied by Shanghai Yunfu Nanotechnology Co., Ltd. (Shanghai, China). Tetrahydrofuran (THF) was supplied by Chengdu Chron Chemicals Co., Ltd. (Chengdu, China). 

### 2.2. Preparation of the Silicone Rubber-Based Ablative Composites

**Figure 1:**
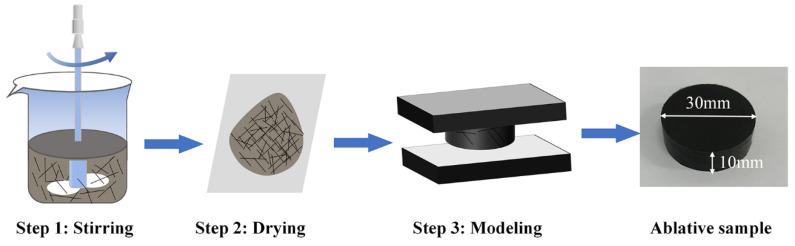
Schematic illustration of the fabrication of ablative samples.

All samples were fabricated by thermal compression molding as shown in Figure 1. Firstly, PDMS, PMPS, and THF were mechanically mixed by overhead stirrers (RW 20 digital, IKA, Staufen, Germany) at room temperature with a mass ratio of 1:1:8 and stirred for 1 h at 300 rpm; then, fillers and vulcanizates were added to the mixture and stirred for 1 h at 300 rpm. To avoid the possibility of destruction of the CFs, the CFs were added last and stirred for 0.5 h for good dispersion. The formula for the silicone rubber-based ablative composites is shown in Table 1. Then, the obtained compounds with CFs of various lengths were dried at room temperature for 12 h to remove all of the THF. Finally, the solid mixtures were vulcanized in a mold at 165 °C and 10 MPa for 10 min. This was followed by a secondary vulcanization process that was carried out at 200 °C for 2 h in an airflow drier.

### 2.3. Characterization

#### 2.3.1. Mechanical and Physical Properties

Dumbbell-shaped samples 2 mm thick were applied for the tensile test using a tensile test machine (CMT-4104, Shenzhen Sans Testing Machine Co., Ltd. (Shenzhen, China).) according to the ISO 37:2005 standard at room temperature (i.e., 25 °C). The tensile rate was 200 mm/min and at least five parallel samples were tested for the average result. The density of composites was tested by a densitometer (GH-120M, Matsuhaku, Xiamen, China) according to the ISO 2781:2007 standard. Sample mass was measured in air and in water marked m_a_ and m_b_, respectively. And the density can be obtained via the formula:

(1) $$\rho =\rho_{0}\frac{m_{a}}{m_{a}-m_{w}}$$

Here, *ρ* and *ρ*_0_ are the density (g/cm^3^) of the materials and water.

Shore A hardness tester (LAC-YJ) was used to measure the hardness of composites in accordance with the ISO 7619:1986 standard, which was provided by Shandong Zhongke Puri Testing Technology Co., Ltd. (Weifang, China).

#### 2.3.2. Ablation Tests

In this study, the ablative properties of silicone rubber-based ablative composites were evaluated by means of an oxyacetylene flame manufactured by Xi’an Zhi Rui Industrial Systems Engineering Co., Ltd. (Xi’an, China). The test standard was according to the Chinese national standard, GJB-323A-1996, and the actual heat flux was 4570 KW/m^2^. The cylindrical sample of Φ 30 × 10 mm was placed vertically at 10 mm from the nozzle tip and ablated for 30 s. The following formulas were derived to calculate the mass and linear ablation rate:

(2) $$R_{m}=\frac{m_{1}-m_{2}}{t}$$

$$R_{l}=\frac{l_{1}-l_{2}}{t}$$

where *R_m_* and *R_l_* represent the mass ablation rate (g/s) and linear ablation rate (mm/s), respectively; *m*_1_ and *l*_1_ are the original mass (g) and thickness (mm) of the samples before testing, *m*_2_ is the mass of composites after ablation, *l*_2_ is the minimum thickness of the ablated samples with the ceramic layer, and *t* is the ablated time (s).

In addition, a K-type thermocouple was applied to the back face of the ablative sample to measure the maximum back-face temperature (T_max, b_) to evaluate the thermal insulation properties. After ablation, the ceramic layer was peeled off and tested for compression properties with a universal testing machine (CMT-4104).

#### 2.3.3. Thermal Stability and Thermal Conductivity Tests

Thermogravimetric analysis (TGA) of cured insulators was carried out from 35 to 800 °C with a heating rate of 10 °C/min under a nitrogen atmosphere, using a thermogravimetric analyzer (TGA2 Iris, METTLER TOLEDO). A transient planar heat source method device (Hot Disk 1500, Hot Disk AB, Gothenburg, Sweden) was used to measure the thermal conductivity of the samples at room temperature.

#### 2.3.4. Morphology Observation

The dispersion of the fibers in the unvulcanized samples was observed by optical microscopy (BX51, Olympus, Tokyo, Japan). The surface and cross-sectional microstructure of the ablated samples were characterized in detail by scanning electron microscopy (SEM, JSM-5900LV, JEOL Ltd., Tokyo, Japan). The crystal phases of the ablated samples were identified by X-ray diffraction (XRD, Ultima IV, Rigaku, Tokyo, Japan), utilizing Cu Kα radiation.

## 3. Results and Discussion

### 3.1. Dispersion, Mechanical, and Physical Properties of the Composites

**Figure 2:**
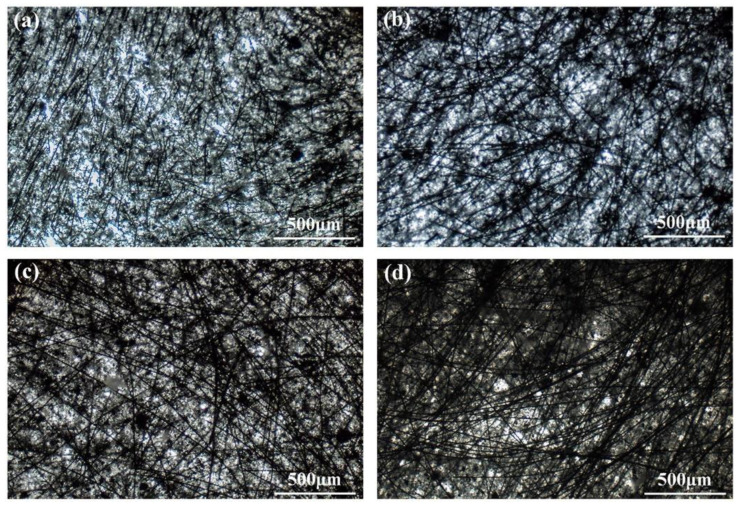
Optical images of unvulcanized samples: (**a**) SF-0.5; (**b**) SF-1; (**c**) SF-3; (**d**) SF-6.

The dispersion and length of the fibers in the composites were the key factors affecting the mechanical properties of the composites. The fibers with a high length-to-diameter ratio had a better enhancement on the composites, which may contribute to improving the ablative resistance. The distribution of the CFs in the composites is shown in Figure 2. The optical images indicate that after a solution mixing process, the CFs were not only maintained their initial length but were well dispersed in the composites. In the SF-0.5 sample, the CFs were partially overlapped only to form a discrete skeleton due to the short fiber length. As the length of the fibers increased, they can make contact and overlap with more fibers; thus, a complete and continuous CF skeleton is formed (Figure 2c,d). Compared with the discontinuous skeleton formed by short fibers, the successive skeleton formed by long fibers (up to 3 mm) can improve the stability of the structure, which may be beneficial for improving the mechanical and ablation properties.

Considering a practical application scenario, a certain strength and elongation should be required for ablative composites to withstand the impact of heat flow and high levels of hot stress. In addition, low density is also desirable for improving the effective payload of motors, just as many density reducers have been filled and studied by researchers. Thus, the mechanical and physical properties of composites with different lengths of fibers were investigated and are presented in Table 2. When the fiber length was 6 mm, the tensile strength and elongation at break were 5.6 MPa and 35.0%, respectively. Although the increase in fiber length can improve the tensile strength of the composites, the elongation at the break of the composites was extremely reduced. Moreover, increasing the length of CFs led to an improvement in the Shore A hardness, while SF-0.5 exhibited excellent flexibility ascribing to the little overlap of CFs. This suggests that longer CFs lose the flexibility of the composites. The density of all composites was no more than 1.3 g/cm^3^, and the variation in density showed the opposite trend with the change in fiber length, indicating large lengths can benefit the higher level of capacity for SRMs.

### 3.2. Thermal Properties of the Composites

**Figure 3:**
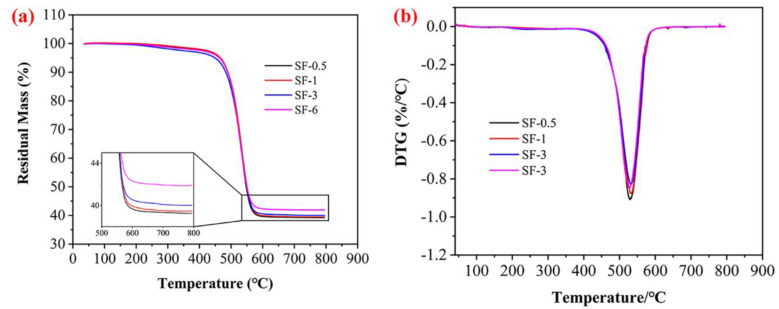
TGA (**a**) and DTG (**b**) patterns of composites under a nitrogen atmosphere.

Figure 3 shows the results of the TGA and DTG referred to composites from 35 to 800 °C in nitrogen. The residue of PDMS and PMPS at 800 °C was less than 10 wt% of the initial mass [13]. In addition, the residual mass of the composites slightly increased with the length growth of the CFs, suggesting that the CFs were stable in nitrogen gas at this temperature. All samples showed a sharp main DTG peak at approximately 520 °C, and it can be associated with the degradation of PDMS and PMPS matrix. It is believed that the length of CFs has little effect on the thermal stability and residual mass of the composites under an inert atmosphere.

### 3.3. Ablation Resistance and Thermal Insulation of the Composites

**Figure 4:**
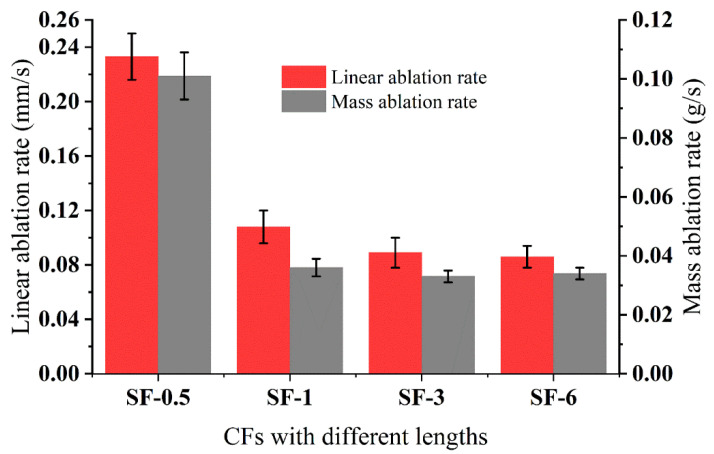
Linear ablation rate and mass ablation rate for the composites with different lengths of CFs.

The linear ablation rate (*R_l_*) and mass ablation rate (*R_m_*) of the composites are illustrated in Figure 4 (the error bars represent the standard deviations). The *R_l_* and *R_m_* of SF-0.5 were 0.233 mm/s and 0.101 g/s, respectively. While increasing the length from 0.5 to 6 mm, the *R_l_* and *R_m_* declined to 0.086 mm/s and 0.034 g/s, respectively. It can be seen that when the length increased from 0.5 to 1 mm, the *R_l_* and *R_m_* decreased by 53.6% and 64.4%, respectively. While the length increased from 1 to 6 mm, *R_l_* only decreased by 20.4%, and the *R_m_* remained almost unchanged. Such variation implies that there is a critical value for the length of CFs to increase the resistance of silicone rubber-based ablative composites. Below the critical length, the resistance of the composites was significantly enhanced by increasing the length. When above the critical length, such enhancement was weakened, which was confirmed by the *R_l_* and *R_m_* of SF-3 and SF-6. That is, a sufficient length of fiber can reinforce the ceramic layer to resist mechanical attack, but thermochemical erosion requires other components to protect and further promote the ablation performance.

The maximum back-face temperature (T_max, b_) can be used to evaluate the thermal insulation of ablative composites. Additionally, the thermal conductivity and ablative resistance of composites were major factors affecting the rate of heat transfer from the combustion chamber to the case. As presented in Table 3 (the ranges defining the scatter of values represent the standard deviations), increasing the length of CFs enhanced the thermal conductivity of the composites, implying the insulation performance would be undermined. In detail, when shifting the length from 0.5 to 3 mm, the T_max, b_ decreased from 117.7 to 107.9 °C. Nevertheless, as the length increased to 6 mm, the T_max, b_ increased to 115.2 °C, which is lower than SF-0.5. Excessive *R_l_* and *R_m_* usually caused the composites to burn through, and the high T_max, b_ caused the engine to not operate properly, so the thermal protection properties of the silicone rubber-based ablative composites were determined by *R_l_* and *R_m_* as well as T_max, b_. As shown in Figure 4 and Table 3, increasing the length of the CFs decreased *R_l_* and *R_m_*, but the T_max, b_ of SF-3 was the optimal one. Taking the mechanical properties of *R_l_*, *R_m_*, and T_max, b_ into consideration, the length of 3 mm is worth considering, and the mechanism of thermal insulation is be discussed in detail in a later section.

### 3.4. Component and Morphological of the Ceramic Layer

**Figure 5:**
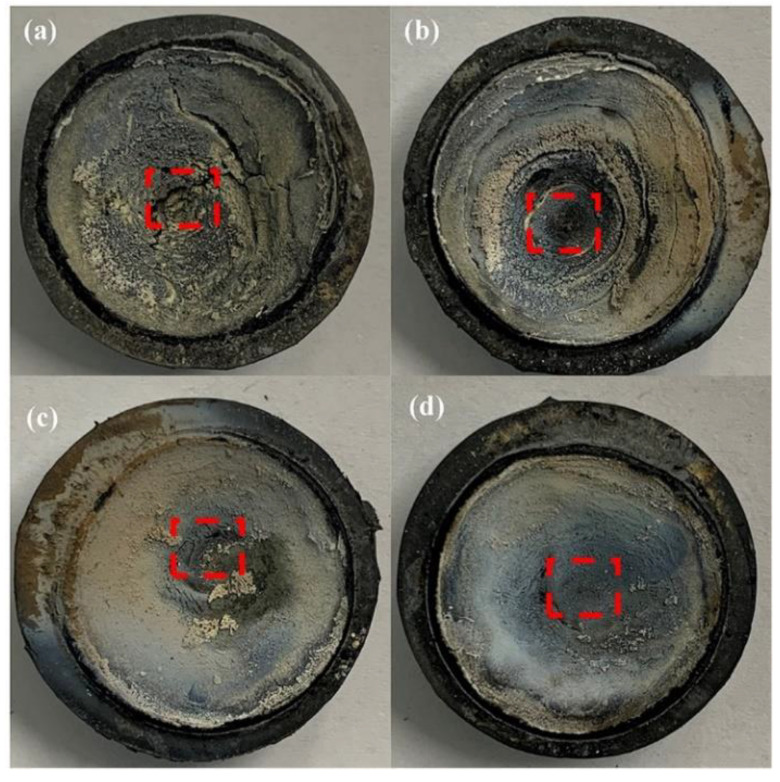
Macroscopic appearance of ablated composites: (**a**) SF-0.5; (**b**) SF-1; (**c**) SF-3; (**d**) SF-6. Red dotted box areas were used to identify crystal phases.

Many studies attribute the diversity of the ablation properties of composites to the different structures of the formed ceramic layers during ablation tests, such as the integrity and denseness as well as strength of the ceramic layer [10,15,16]. The macroscopic appearance of the ablated surface is shown in Figure 5, and different shapes of pits were observed on the sample after ablation. In addition, the ceramic layer of SF-0.5 showed obvious cracks, indicating weak strength and poor interaction between the ceramic layer and virgin materials, and increasing the length of CFs improved the compactness and integrity of the ceramic layer (Figure 5c,d). Moreover, the surfaces of all ablated composites were covered with an off-white material, probably a mixture of silica and zirconium dioxide (an oxidation product of zirconium boride).

**Figure 6:**
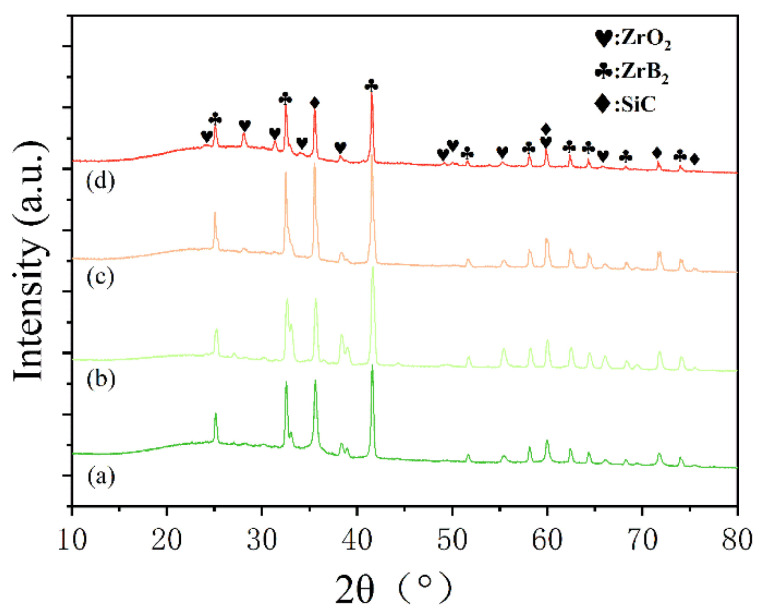
XRD patterns for the ablated surfaces of the composites: (a) SF-0.5; (b) SF-1; (c) SF-3; (d) SF-6.

The red, dotted box areas in Figure 5 of the ablated composites were peeled and identified by XRD as shown in Figure 6. There were three distinct XRD peaks at approximately 35.9°, 60.5°, and 71.9° that matched to (111), (220), and (311), indicating the presence of SiC [32]. In addition, SiC may be produced by the reduction reaction of C and SiO_2_ at high temperature. Moreover, the characteristic peaks of SiO_2_ (PDF 65-0466) did not appear due to the melting point of SiO_2_ (1723 °C) being much lower than the temperature of the oxyacetylene flame (3000 °C), and the molten SiO_2_ could be easily blown away by high-speed heat flux because of its low viscosity. Furthermore, the weak characteristic peaks of ZrO_2_ (PDF 37-1484) formed by the oxidation of ZrB_2_ were observed only in SF-3 and SF-6 [33]. Compared to SiO_2_, the melting point of ZrO_2_ (up to 2700 °C) was close to the temperature of the oxyacetylene flame. Although most of the ZrO_2_ was carried away by the high-speed heat flux to provide heat dissipation, and the successive skeleton formed by longer CFs (more than 3 mm) played a fixed role, allowing the ZrO_2_ to stay on the ablated surface for a long time, allowing for observation.

**Figure 7:**
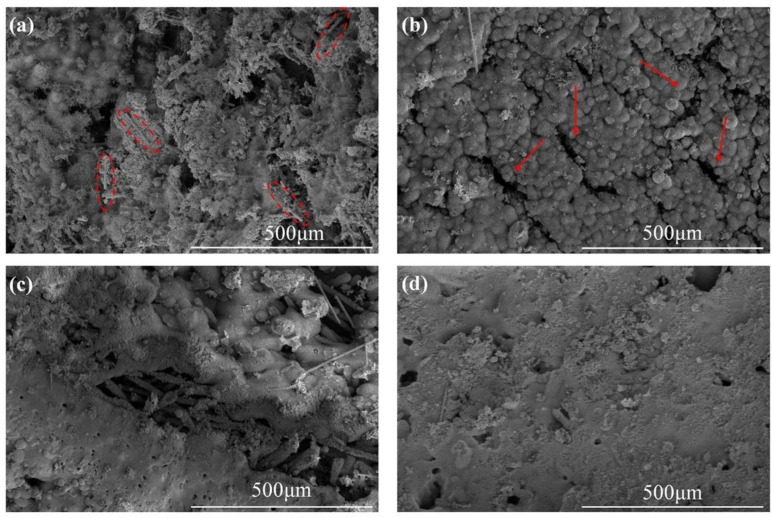
SEM images of the ablated surfaces: (**a**) SF-0.5; (**b**) SF-1; (**c**) SF-3; (**d**) SF-6. The red oval areas represent exposed CFs after ablation. The red arrows denote cracks after ablation.

**Figure 8:**
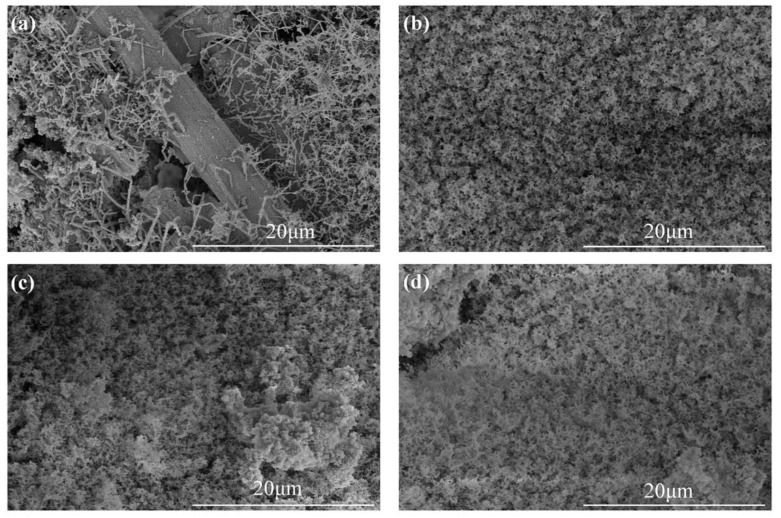
High magnification SEM images of ablated surfaces: (**a**) SF-0.5; (**b**) SF-1; (**c**) SF-3; (**d**) SF-6.

The SEM images of the ablated surfaces displayed in Figure 7 and Figure 8 were used to study the effect of CFs on the ablation process in detail. As shown in Figure 7a, several holes on the irregular ablated surface of SF-0.5 were found, and some CFs (red oval areas) were exposed. From the high magnification SEM image (Figure 8a), it can be seen that there was no obvious interaction between the residue of silicone rubber pyrolysis or fillers and the CFs. Although there were no pits and micro-cracks on the surface of CFs after ablation, the reinforcing effect of CFs for the ablated surface was not shown. Moreover, the ablated surface of SF-0.5 with such characteristics cannot effectively shield the heat flux and the erosion of oxidizing gas. Hence, the ablated surface of SF-0.5 lacked sufficient reinforcement by CFs and could not withstand the thermo-mechanical erosion, resulting in loss of integrity (Figure 5a) and poor ablative resistance (Figure 4). As shown in Figure 7b, the relatively flat ceramic layer with obvious cracks (red arrows) was covered in the ablated surface of SF-1 and had no CFs exposed. Due to the pyrolysis of the matrix and the melting and evaporation of the fillers, only CFs can maintain the virgin structure in such a harsh environment. From the result of Figure 2b, it is known that a relatively continuous skeleton was formed, which played a significant role in fixing residues and fillers during the ablation process. Therefore, compared with SF-0.5, *R_l_* and *R_m_* were greatly reduced, and the ablation mechanism was necessarily different. With the further increase in the length, the continuity of the ceramic layer formed on the ablated surface of SF-3 and SF-6 improved, but there were still a small number of pores on the ablated surface (Figure 7c,d). At this time, the ceramic layer with a complete surface could resist the impact of heat flux and the erosion of oxidizing gas, so that the protection for the internal composites is more effective. In this work, the skeleton role has been reflected when the length of the fibers was greater than 1 mm; however, the further improvement of ablative resistance was limited by the low fillers loading (Figure 4).

**Figure 9:**
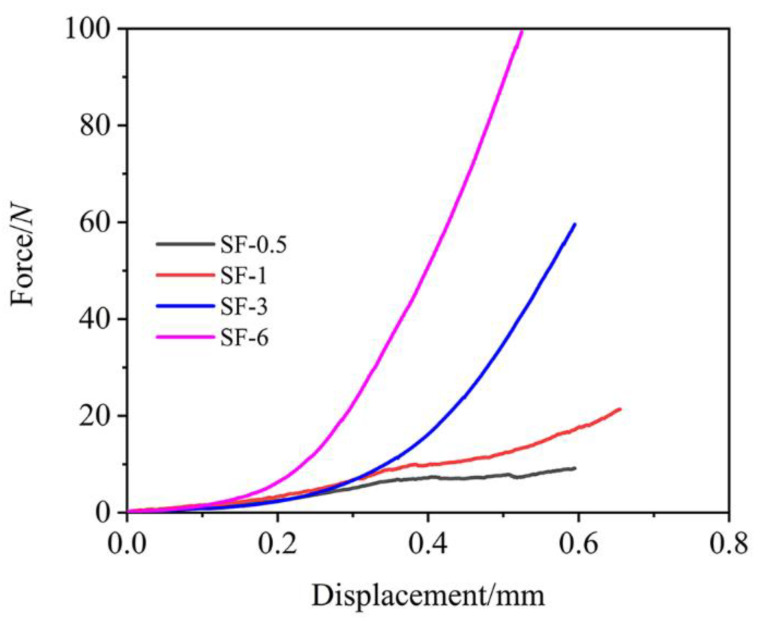
Compressive performance of the ceramic layer.

The excellent compression resistance of the ceramic layer makes an outstanding contribution to the resistance of particle or heat flow erosion. The ceramic layer was peeled off, and the compression test results are illustrated in Figure 9. It can be easily seen that fiber length had a significant effect on the compressive properties: increasing the length promoted compressive resistance. It is noteworthy that unsmooth displacement–force curves for SF-0.5 and SF-1 were observed, while the curves for SF-3 and SF-6 were not only smooth but also had much better compression resistance than SF-0.5 and SF-1, which corresponded to the results of *R_l_* and *R_m_*.

### 3.5. Ablation Mechanism of the Composites

In silicone rubber-based ablative composites, the matrix was completely decomposed at approximately 600 °C, with very low residue content and no strength. During oxyacetylene ablation, the residue had no thermal protection capabilities after being washed by heat flux and the thermochemical corrosion of oxidizing. Although the ceramic fillers layer with a high melting point can be melted to form a liquid ceramic layer and absorb lots of heat, its low viscosity makes it easy to be blown away without the support of the skeleton. Accordingly, the silicone rubber-based composites have limited ablation resistance. CFs have a high degree of graphitization, and their thermal stability and residual strength at high temperatures were significantly higher than that of the matrix. Therefore, CFs can serve as a connecting phase to fix the residue and liquid ceramic layers.

**Figure 10:**
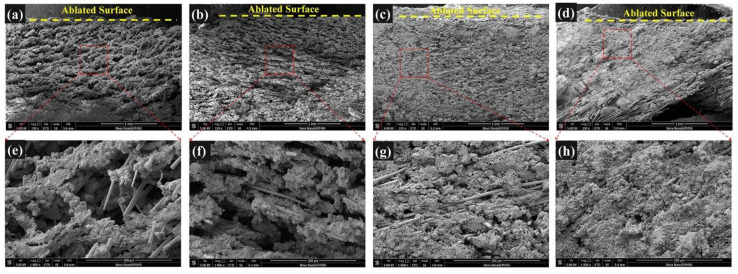
Cross-sectional microstructure of ceramic layer: SF-0.5 (**a**,**e**); SF-1 (**b**,**f**); SF-3 (**c**,**g**); SF-6 (**d**,**h**).

**Figure 11:**
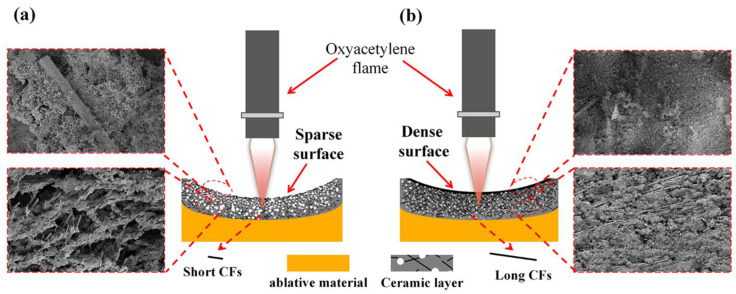
Ablation mechanism of CF-filled silicone rubber-based ablative composites: (**a**) short carbon fibers, (**b**) long carbon fibers.

The cross-sectional structure of the ceramic layer of the ablation was characterized by SEM to analyze the ablation mechanism in detail, and the results are shown in Figure 10. For SF-0.5, numerous holes inside the ceramic layer were observed, and many CFs were exposed (Figure 10a,e), which is consistent with the ablated surface. Moreover, the integrity and strength of the ceramic layer were poor due to the insufficient reinforcing effect of CFs with short length on the ceramic layer (Figure 7a and Figure 9). Therefore, it can be inferred that the main reason for the poor ablation performance of SF-0.5 was the weak interfacial interactions and that the local skeleton was unable to transfer the forces generated by ablation to the whole ceramic layer, thus causing the ceramic layer to break down (as shown in Figure 11a). For SF-1, the ablation holes inside the ceramic layer were reduced, and the integrity of the ceramic layer was better (Figure 10b,f). Because the fibers were long enough, they could take advantage of their length to concatenate and consolidate the ceramic fillers to form a relatively complete ceramic layer. Thus, the residue and the molten fillers could survive on the ablated surface of the ceramic layer for a longer time rather than be entirely blown away, showing favorable resistance to oxidizing gas and high-speed heat flux. With further growth of the CFs length, the inside of the ceramic layer tends to be extremely dense, and the consolidation of CFs are better reflected (Figure 10c,d). Under the action of the successive skeleton formed by long CFs, the ablation resistance of silicone rubber-based composites is primarily determined by the ceramic layer on the ablated surface to endure oxidizing gas and high-speed heat flux (shown in Figure 11b). Here, the increase in length of CFs from 3 to 6 mm with a slight improvement in *R_l_* and *R_m_* has been adequately explained.

**Figure 12:**
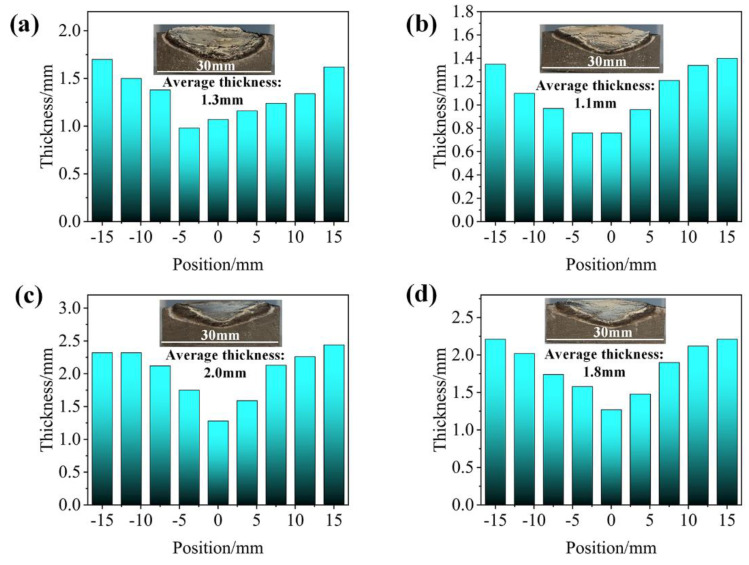
Thickness distribution of the ceramic layer: (**a**) SF-0.5; (**b**) SF-1; (**c**) SF-3; (**d**) SF-6.

Thermal insulation mainly involves thermal conductivity and external heat flux. The thickness of the ceramic layer was measured, and the average thickness of the ceramic layer was statistically obtained as shown in Figure 12. It can be seen that the maximum thickness of the ceramic layer of SF-3 was 2.0 mm and the thinnest layer of SF-1 was 1.1 mm, which proves that the increase in fiber length improved the erosion resistance of the ceramic layer. There was variability in the T_max, b_ for different CFs lengths due to the following reasons. On the one hand, the increase in the length of fibers led to a corresponding increase in the thermal conductivity of the virgin composites (Table 3), resulting in the promotion of endothermic and caramelization of silicon rubber to improve the efficiency of ceramic formation. On the other hand, the ceramic (residue and fillers) on the ablated surface can better shield the heat flux when heat flux invades composites; the ceramic layer structure of SF-0.5 had limited erosion resistance causing high *R_l_* and *R_m_* (Figure 4); thus, this T_max, b_ was the highest. SF-3 had the most satisfactory ability to resist thermomechanical erosion due to the skeleton formed by CFs, while the proper porous structure within the ceramic layer and the thickest ceramic layer (Figure 10d,h) had excellent thermal insulation properties. The thickness and thermal conductivity of the ceramic layer were what limited the insulation performance of SF-6, even though it had the best ablation resistance.

## 4. Conclusions

In this work, CFs with different lengths were introduced in silicone rubber-based ablative composites, and the effect of fiber length on ceramic layer morphology and ablative properties were systematically studied. The linear ablation rate declined from 0.233 to 0.089 mm/s, and the maximum back-face temperature decreased from 117.7 to 107.9 °C, as the length of CFs increased from 0.5 to 3 mm. Morphological study of the ceramic layer demonstrated that by taking advantage of the appropriate length (3 mm), CFs can concatenate and consolidate the ceramic fillers and residues to form an integrated and robust as well as dense ceramic layer. In addition, the thick ceramic layer and low thermal conductivity help to improve thermal insulation performance. As for the effective utilization of carbon fibers, this work has obvious advantages and great potential in building ablative skeletons to develop composites with excellent ablation and insulation properties that have promising applications in thermal protection systems and beyond.

## Figures and Tables

**Table 1:** Composition of the samples (part per hundred grams silicone rubber).

Samples	PDMS	PMPS	DBPMH	SiO_2_	ZrB_2_	B_4_C	CFs
SF-0.5	50	50	0.5	30	17.5	2.5	10, 0.5 mm
SF-1	50	50	0.5	30	17.5	2.5	10, 1 mm
SF-3	50	50	0.5	30	17.5	2.5	10, 3 mm
SF-6	50	50	0.5	30	17.5	2.5	10, 6 mm

**Table 2:** Mechanical and physical properties of the composites.

Samples	Tensile Strength (MPa)	Elongation at Break (%)	Hardness (Share A)	Density (g/cm^3^)
SF-0.5	3.30 ± 0.06	421.9 ± 7.9	78.7 ± 1.1	1.281 ± 0.002
SF-1	4.06 ± 0.37	224.3 ± 5.6	82.9 ± 1.6	1.279 ± 0.004
SF-3	4.39 ± 0.50	184.1 ± 5.9	85.7 ± 1.4	1.275 ± 0.006
SF-6	5.60 ± 0.95	35.0 ± 9.8	89.8 ± 1.1	1.267 ± 0.004

**Table 3:** The maximum back-face temperature and thermal conductivity of the composites.

Samples	T_max, b_ (°C)	Thermal Conductivity (W/mK)
SF-0.5	117.7 ± 0.9	0.3089 ± 0.0011
SF-1	109.0 ± 1.5	0.3178 ± 0.0035
SF-3	107.9 ± 1.1	0.3211 ± 0.0052
SF-6	115.2 ± 1.8	0.3401 ± 0.0038

## Data Availability

Data from this study are available upon request from the corresponding authors.
